# International accreditation for acute stroke care: lessons learnt from a Kenyan Stroke Centre

**DOI:** 10.3389/fstro.2025.1599649

**Published:** 2025-09-02

**Authors:** Dilraj Singh Sokhi

**Affiliations:** Department of Medicine, Faculty of Health Sciences, Medical College of East Africa, Aga Khan University, Nairobi, Kenya

**Keywords:** stroke, care pathway, low resource settings, sub-Saharan Africa, ischemic stroke, hemorrhagic stroke

## Abstract

**Background:**

The prevalence of stroke is increasing in Africa, yet resources remain limited in managing the disease. Whilst there are international guidelines on how to set up and manage stroke services, even in resource-limited settings, the uptake remains low. We describe here the opportunities and challenges we faced whilst setting up a stroke care pathway of international standards in a regional referral hospital in East Africa.

**Methods:**

We describe how we adapted international stroke care guidelines for acute primary stroke (including both ischemic and hemorrhagic stroke), and used these to inform our stroke care pathway. We highlight opportunities of leveraging on multi-disciplinary involvement, as well as challenges of implementing the pathway.

**Results:**

Our hospital was accredited by the Joint Commission International with a Clinical Care Programme Certification in May 2021. However, there were strategic improvement plans recommended that needed to be addressed for future re-accreditations, including having a dedicated stroke unit and addressing shortfalls in thrombolysis and thrombectomy timelines. We discuss the challenges faced with these and other relevant findings from the accreditation process.

**Conclusion:**

International accreditation of our hospital provides an example of how to adapt international guidelines to local contexts. The description of our experience may be useful for other healthcare institutions from resource-limited settings who strive to improve the quality of stroke care they provide.

## Introduction

Stroke incidence is increasing in Africa due to a complex interplay of increasing non-communicable diseases, lifestyle factors as well as communicable disease in Africa (Abissegue et al., 2024). Stroke incidence is increasing in Kenya too, and the mortality rates remain very high (Kaduka et al., 2018). Factors contributing to these poor outcomes are patient-related (lack of knowledge about symptoms and treatments for stroke), infrastructural (inadequate or poor emergency and primary healthcare systems), and due to poor resources (no stroke centers in the country, and a severe dearth of specialist healthcare workers). Stroke centers are known to improve outcomes for patients, yet none specifically exist in East Africa (Roushdy et al., 2022). The first comprehensive stroke care guidelines for the country were only launched in 2024 through the 2nd edition of the National Cardiovascular Disease management guidelines, but implementation has been slow due to systemic lethargy (Orangi et al., 2023).

The Medical Board in Kenya defines secondary care hospitals as being Level 5 or Level 6 healthcare facilities, defined by their ability to provide specialized services as a referral center, as well as engagement in clinical research and providing medical training. Over a third of secondary healthcare in Kenya is provided by the private sector (Lee et al., 2022), and The Aga Khan University Hospital (AKUHN) is one of the main private level 5 referral hospitals in the country. AKUHN is unique in that it is the only private not-for-profit secondary-level healthcare institution that also undertakes academic roles, including clinical research and provision of training for undergraduates and post-graduates in most cadres of the healthcare workforce.

In 2013 it was the first hospital in the region to attain Joint Commission International Accreditation (JCIA) for maintaining adequate standards of healthcare. Within the JCIA framework are disease-specific accreditations called Clinical Care Programme Certifications (CCPC), and in 2020 AKUHN was CCPC-accredited for providing world-class healthcare for acute myocardial infarction (AMI).

Initiatives within the hospital had already established some elements of a stroke system e.g., having stroke-specific protocols in our emergency department, and the regular provision of thrombectomy services (AKUHN, 2018). Our hospital therefore strove to next apply for CCPC accreditation for stroke care. In this article, we highlight the journey toward international accreditation, the challenges faced, and discuss how other hospitals in the region can learn from our experience.

## Methods

### Building a relevant framework

There is a relatively higher incidence of hemorrhagic stroke in sub-Saharan Africa (SSA), probably due to the higher prevalence of hypertension Given the phenotypes of ischemic and hemorrhagic stroke are similar, and differentiation is only after neuro-imaging, it made sense for all patients with stroke symptoms to be admitted under a unified stroke care pathway for both types of stroke. We therefore aimed to produce a care pathway for Acute Primary Stroke, which included both ischemic and hemorrhagic types. Other causes of acute cerebrovascular disease (subdural hematomas, subarachnoid hemorrhage and cerebral venous sinus thrombosis) were excluded.

### Guidelines and key performance indicators

We were fortunate to have access to international guidelines for stroke care from the developed world, and had the liberty to choose the ones that would most be suited to our hospital. There were some differences between noted between some guidelines; e.g., the European and the American guidelines differed on what level of carotid stenosis would require intervention (Mulder et al., 2024). Whilst our cardiology team adapted European guidelines for the AMI programme, we opted to adapt the American guidelines (Powers et al., 2019; Greenberg et al., 2022), as most of our team were familiar with them and used them in the day-to-day care of stroke patients already.

For both types of stroke, we tabulated the recommendations from the respective guidelines: from the 2019 ischemic stroke guidelines we had 262 items, and from the 2015 and 2022 intracranial hemorrhage guidelines we had 150 items (see Supplementary material). We went through each item and first decided whether it was relevant in our context, and if so if it needed any adaptation for implementing in our hospital. For example, in ischemic stroke, item 1.6.1. talks of using tele-radiology to report scans in the US, but is not available in Kenya so we discarded this item. However, item 6.3.4. suggests routine echocardiography is not necessary as it may not be economical, but we adapted this to saying we would do echocardiography in all patients as the service is readily available given the AMI accreditation. All these items were then allocated to specific team leads within the care pathway, who were charged with ensuring their implementation.

The next step was to identify key performance indicators (KPIs). Whilst the guidelines provided most of these, there were some that were beyond our reach in the initial stages (e.g., imaging done within 20 min of arrival), so we instead adapted the KPI from outside the guidelines; e.g., the total percentage of patients who undergo neuro-imaging, taken from the World Stroke Organisation (WSO) toolkit (Saposnik et al., 2022). We selected KPIs that reflected the breadth of the journey of the stroke patient in our hospital, including after discharge. It was therefore important to also include the American guidelines for the allied specialties including physiotherapy and speech therapy for stroke into our master guideline (Winstein et al., 2016).

**Figure 1:**
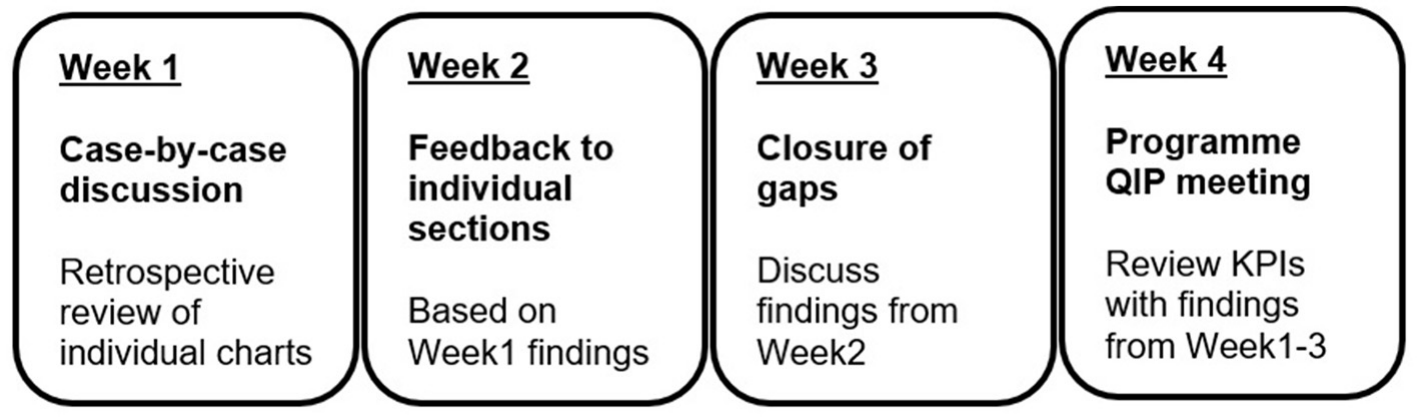
Programme quality improvement process (PQIP).

We additionally set up a monthly programme-specific quality improvement process (PQIP) meeting to complement the department- and hospital-wide QIPs, so as to have an iterative process of improving on our care as measured through the KPIs. This stroke QIP is attended by all section leads, and additional healthcare workers are invited as needed e.g., to discuss aspects of a specific patient's care (see Figure 1).

### Pre-hospital care: a big exclusion

Unfortunately, we had to completely exclude the entire first sections of the guidelines which focused on pre-hospital emergency response systems. Whilst Kenya is attempting to provide universal health coverage for all its population, there has been very little progress toward this, particularly in the provision of emergency services including ambulances (Wachira and Mwai, 2021; Lee et al., 2022). At present the majority of ambulances are provided by private companies or faith-based services, and even though there are national emergency numbers, there is no guarantee that appropriate services will be deployed in a timely manner especially outside urban settings (Wachira and Mwai, 2021). Given AKUHN does not have its own ambulance service either, we decided to not include pre-hospital care in our care programme.

We did, however, embark on awareness campaigns, and joined efforts with the Stroke Organisation of Kenya to increase public awareness about stroke and the treatments made available (Wairimu, 2018). We also investigated infrastructural and knowledge barriers to stroke in a prospective study (Mithi et al., 2021), and used these findings to inform our public health information campaigns.

### Guideline to care pathway: importance of international partnerships

We needed to now crystallize the different sections of our master guideline into a user-friendly care pathway document so that front-end healthcare workers could easily provide the evidence-based care for our stroke patients. We went back to the item-by-item list and whatever items were actions, we listed them into the care pathway document. The respective sections were then reviewed with the users from each section of the hospital and adjustments made, and then we trained all members of the healthcare team in line with our master guideline and the care pathway document.

In addition to the above process, we were fortunate to have the Angels Initiative reach out to our hospital to help make it stroke-ready, and in this regard it facilitated helpful exchange programmes (Correspondent, 2018). For example, we visited world-class stroke centers in Dubai for knowledge exchange, and specialist nurses from South Africa held a workshop in Kenya to upskill our critical care nurses. In this way we could learn from other institutions to fashion our own stroke care programme.

### Challenges: personnel, COVID, cost

Multi-disciplinary team (MDT) involvement at the outset was important not only for the purposes of training all team members but also to highlight deficiencies in the system. We realized quickly that our part-time speech therapist would be overwhelmed with daily stroke reviews, and we had to recruit another therapist from the pool of only 30 in the country. We also noted there were no specialized neuro-rehabilitation physiotherapists in the country including at AKUHN, and ended up doing online upskilling in collaboration with neuro-physiotherapists from University of California, San Francisco (Block, 2020).

Importantly, there are only two neurovascular interventionalists in the country, one of whom works at AKUHN (Mogere et al., 2022); we had to consider how to fill the service gap and even entertained training one of our interventional cardiologists, although this approach remains controversial (Hornung et al., 2019). In the end, we chose to only enlist the two appropriately credentialed neuro-interventionalists to provide thrombectomy services at our hospital.

It took over a year to get us to the stage of rolling out the programme, but then the COVID-19 pandemic hit our country, and just as was experienced in other parts of the world we had delays in delivering and implementing stroke services (Wu et al., 2020). We noted huge delays in patients getting to hospital, and within the hospital triaging system, and a number missed time windows for acute interventions such as thrombolysis as a result: our thrombolysis rates halved from 2.36/month to 1.2/month (Sokhi, 2021).

Setting up stroke services is not a cheap endeavor; in the US the investment required can be close to $1 m over 3 years for a single center (Yarnoff et al., 2019). To keep costs to a minimum, we had to identify building blocks that already existed within our hospital ecosystem that we could then bring together to inform the stroke pathway. Nevertheless (as detailed above), we still had to spend to enroll new personnel in most if not all sections of the care pathway to ensure consistent delivery. In particular, our quality department had to dedicate a specific team of staff members (clinical nurse, education lead, data enumerator and statistician, administrative support) toward the stroke pathway. A significant but unmeasurable indirect cost was the administrative time (data meetings, review of protocols etc.) spent by the members of our team outside their usual clinical work toward getting the pathway off the ground.

## Results

### Accreditation and after

Despite the challenges, we managed to fulfill the criteria for CCPC accreditation in May 2021, 3 years from conceptualization. However, there were a few caveats: foremost, we did not (and still do not) have a dedicated physical space called a stroke unit, which is Class I evidence for improving stroke care in any setting (Powers et al., 2019). As a moderate-sized hospital of 330 beds, we provide an equivalent moderate-sized stroke service with ~100 admissions a year. Guidelines e.g., from Australia suggest that if a hospital admits more than 75 patients a year with stroke, it should have a dedicated stroke unit (SFA, 2019). Nevertheless, we navigated around these Internal barriers (due to cost and space limitation) by having the stroke care housed within our critical care unit, where we ensured all staff were appropriately trained to look after acute stroke admissions.

Advertising ourselves as an accredited stroke center has increased the number of patients referred especially from outside Nairobi city and outside the country. However, it was important for us to know if achieving world-class status in stroke has translated to a difference in outcomes. It is interesting to note that the respective KPIs such as door-to-needle time, door-to-puncture time, length of stay and mortality rates all remained largely unchanged when comparing pre- and post-accreditation cohorts (Sokhi et al., 2024), and we have continued to struggle to meet the treatment-specific KPIs. These findings can be viewed as bittersweet: perhaps we were already doing things so well before accreditation, or the effects of the pandemic diluted the positive impact of our programme. Pre-hospital factors remain a big factor which we cannot address (as described earlier in this article): a third of patients still arrive late (>24 h of onset), emergency services remain suboptimal, and other hospitals have been slow to improve their referral and triage system to our unit.

## Discussion

### About hyper-acute treatments

The struggle to make a tangible improvement in our hyper-acute treatment KPIs (thrombolysis and thrombectomy timelines) are more a reflection on how we continue to manage the care pathway, rather than a gap in the accreditation process. The JCIA surveyors looked keenly at improvement in our performance enhancement measures rather than focusing on absolute percentages, and this in itself is an indication of a programme that strives to self-improve. For example, in the recertification survey in 2024 it was noted that whilst we were still not meeting these treatment KPIs, we had strong evidence of a trend toward improvement compared to 2021. Internally, one of the major factors that impedes achieving our KPIs is high staff turnover, not only within front-end workers but also within leadership and administrative positions. We have realized that good organizational memory is critical toward ensuring our improvement initiatives are sustained and re-evaluated rather than simply recirculated. Harnessing our hospital's computer infrastructure has been one of our major solutions to maintain organizational memory:

- use of shared online drives for referring to documents/protocols/minutes;
- easily accessible online training videos made specifically for each different section of the pathway;
- direct migration of the care pathway to our world-class electronic health record system that was deployed in late 2022.

### Comparison to similar initiatives in the region

Striving for international recognition for provision of quality care improves patient outcomes and therefore an aspiration dear to perhaps many hospitals in the region. However, the level to which this is achieved is limited by local resources and expertise. As a private hospital, we are fortunate to be able to provide the latest that is available in terms of diagnostics and treatment, but that should not necessarily make us a better quality hospital. It is interesting to see what was done e.g., in Tanzania to improve stroke care at two large public hospitals (Matuja et al., 2025). The authors employed very practical steps to measure their stroke burden (set up stroke registries), upskill their personnel (online modules from the Angels Initiative/WSO with pre- and post-assessments), and inform their stroke unit care pathway protocols (using other regional guidelines from Ethiopia and Zambia). The success of their policy-driven interventions was due to multi-stakeholder involvement at all levels of the public healthcare system feeding into their hospitals. All these are important steps that perhaps we could have followed to make our adaptation of American guidelines easier in the local context. However, notably the Tanzanian studies did not have thrombolysis or thrombectomy available at either hospital, reflecting the resource limitation for these particular KPIs that we can provide routinely in our hospital.

We accept that, being a private hospital, we have inherent differences compared to public hospitals in the region which could make it difficult to generalize our experience in setting up a stroke service. However, public-private partnerships are important in helping institutions to learn from each other to improve health systems on the continent (Oleribe et al., 2019), and in this regard AKUHN does have a role in collaborating with the Kenyan public health system especially when it comes to providing universal health coverage (MOH, 2023). As the efforts led by AKUHN faculty help standardize emergency care in the country continue (Lee et al., 2022), perhaps our stroke service implementation will likewise resonate with the public hospitals in years to come. Indeed, in Zambia international stroke care guidelines, including from the AHA/ASA which we used for our care pathway, were successfully adapted and implemented in a resource-limited public referral hospital (Shumba et al., 2024).

## Conclusion

Overall, our experience in undergoing the CCPC accreditation was positive. The endeavor has fostered a spirit of teamwork, and all members of the care pathway feel accountable to the programme. There is more cross-departmental collaboration to ensure we deliver on good stroke care. Even in the face of shortage of specialists or facilities, we have leveraged on our strengths to be innovative in how we deliver the care.

We realized we focused more on the technicalities of the stroke care pathway (adaptation of guidelines, training of personnel) but perhaps forgot the more people-centric and holistic approach adopted by other stroke centers to get better buy-in and involvement from all healthcare workers (Reckless et al., 2008): forging partnerships and being more engaging with senior leadership are some examples of where we could have done better.

We envisage that as the programme matures, we will continue to see positive changes in the population that we serve in the long-term, whilst reflecting on the state of our care in the medium term to help inform future directions. Ours is an example that we can provide world-class stroke care in a resource-limited setting, and we encourage our colleagues in the region to consider following a similar remarkable journey.

## Data Availability

The data analyzed in this study is subject to the following licenses/restrictions: Original data can be requested from the author. Requests to access these datasets should be directed to dilraj.sokhi@aku.edu.
